# Taking a holistic approach to managing difficult stress fractures

**DOI:** 10.1186/s13018-016-0431-9

**Published:** 2016-09-09

**Authors:** Timothy L. Miller, Thomas M. Best

**Affiliations:** 1 Orthopaedic Surgery and Sports Medicine, The Ohio State University Wexner Medical Center, Columbus, OH USA; 2 Capital University Athletics, 920 North Hamilton Road, Suite 600, Gahanna, OH 43230 USA; 3 Academic Affairs, Department of Family Medicine, The Ohio State University Wexner Medical Center, Columbus, OH USA; 4 Biomedical Engineering, The Ohio State University, Columbus, OH USA

**Keywords:** Bone, Fracture, Insufficiency fracture, March fracture, Overuse injury, Stress fracture, Stress injury, Stress reaction, Stress response, Tibia

## Abstract

Stress fractures and other bony stress injuries occur along a spectrum of severity which can impact treatment and prognosis. When treating these injuries, it should be borne in mind that no two stress fractures behave exactly alike. Given that they are not a consistent injury, standardized treatment protocols can be challenging to develop. Treatment should be individualized to the patient or athlete, the causative activity, the anatomical site, and the severity of the injury. A holistic approach to the treatment of the most difficult stress fractures should be taken by orthopedists and sports medicine specialists. This approach is necessary to obtain optimal outcomes, minimize loss of fitness and time away from sports participation, and decrease the risk of recurrence.

## Background

### The holistic approach

Stress fractures occur along a continuum of severity and may occur in nearly any sports or repetitive activity [1]. Certain sports are more commonly associated with stress fractures, including running (69 %), fitness class/cross-fit (8 %), racket sports (5 %), and basketball (4 %) [2]. In order to optimize a patient’s recovery and outcome from these injuries, a holistic approach should be taken by orthopedists and sports medicine practitioners that includes specialists in athletic training, nutrition, endocrinology, psychology, sports-specific mechanics, and physical therapy.

Treatment principles for stress fractures include re-establishing the normal balance between the creation and repair of microcracks in the bone [3]. In order to decrease this repetitive microtrauma, providers must evaluate the patient’s training regimen, biomechanics, and equipment. Maximizing the patient’s biologic capacity to repair microcracks requires an assessment of the athlete’s general health focusing on nutritional behaviors, hormonal status, and medication and tobacco use [4].

## Pathophysiology

Healthy bone is in constant homeostasis between microcrack creation and repair. Fatigue failure of the bone has three stages: crack initiation, crack propagation, and complete fracture [1, 5]. Crack initiation typically occurs at sites of stress concentration during bone loading. Crack propagation occurs if loading continues at a frequency or intensity above the level at which new bone can be laid down and microcracks repaired. Continued loading and crack propagation allows for the coalescence of multiple cracks to the point of becoming a clinically symptomatic stress fracture. If the loading episodes are not modified or the reparative response is not increased, crack propagation can continue until structural failure or complete fracture occurs [5].

## Risk factors for developing a stress fracture

A variety of biological and mechanical factors are thought to influence the body’s ability to remodel bone and therefore impact an individual’s risk for developing a stress fracture. These include but are not limited to sex, age, race, hormonal status, nutrition, neuromuscular function, and genetic factors [6]. Other predisposing factors to consider include abnormal bony alignment, improper technique/biomechanics, poor running form, poor blood supply to specific bones, improper or worn-out footwear, and hard training surfaces. It is important to remember that the cause of stress fractures is multifactorial, and the list of differential diagnoses is extensive [4, 7] (Table 1).

**Table 1:** Differential diagnoses for stress fractures

Tumor Infection Sickle cell disease Medial tibial stress syndrome Chronic exertional compartment syndrome Nerve and arterial entrapment Metatarsalgia	

### Neuromuscular hypothesis

Muscle strength can also affect an individual’s susceptibility to stress fractures. Proper neuromuscular function can dissipate the energy of externally applied impact loads on the bones and joints that can occur during running and jumping. Muscle fatigue may be an important factor in fatigue fractures [8]. This is referred to as the *neuromuscular hypothesis* [1, 4]. As muscle fatigues, its capacity to absorb the energy of an externally applied load diminishes, resulting in higher peak stresses and more rapid accumulation of microdamage [8]. Overall, general fitness is protective, and studies have shown that military recruits with higher activity levels before enlistment had fewer stress fractures during basic training [8].

### Overtraining syndrome

Overtraining has been a recognized cause of injury since the ancient Greek Olympic games. Endurance sports training require a balance between workload and recovery. Athletes such as competitive runners and triathletes often exercise longer and harder in order to improve performance, but work overload and too little time for recovery may lead to physical and psychology symptoms of overtraining syndrome [9]. This condition frequently occurs in athletes who are training for competition or a specific event and train beyond the body’s ability to recover [10]. The muscle fatigue and repetitive impact on hard training surfaces increases the athlete’s risk of developing stress fractures. Without adequate rest and recovery, overly aggressive training regimens increase the risk of injury, cause negative feelings for the activity and those involved in the training, and paradoxically decrease athletic performance [10]. Rest, adequate hydration and caloric intake, and varying the training program with cross-training activities are the mainstays of recovery [9].

### Vitamin D insufficiency

Recent studies have evaluated the potential association between serum vitamin D levels and stress fractures [11]. A prospective study of Finnish military recruits found that the average serum vitamin D concentration was significantly lower in the group that had sustained a stress fracture [12]. Another randomized, double-blind, placebo-controlled study examined whether calcium and vitamin D intervention could reduce the incidence of stress fractures in female recruits during basic training [12]. This level 1 study suggests that calcium and vitamin D supplementation may have prevented a significant percentage of their recruits from sustaining a stress fracture along with a significant decrease in morbidity and financial burden [12].

It is recommended that most patients should receive 800–1000 IU (or perhaps as much as 2000 IU) of vitamin D3 daily because it is relatively safe and has a high therapeutic index. Serum 25(OH)D_3_ level is the study of choice for identifying vitamin D deficiency [13]. In those individuals with low vitamin D or low bone mineral density, the therapeutic goal for supplementation should range from at least 50 nmol/L (20 ng/mL) to as high as 90–100 nmol/L (36–40 ng/mL) based on the Food and Nutrition Board recommendations [13]. Although higher dietary intake of vitamin D may provide some protective effect against fractures, the exact role of vitamin D in fracture prevention is still up for debate.

### Caloric insufficiency and the female triad

Inadequate caloric intake may play a role in amenorrhea, which has been linked to an increased incidence of stress fractures. Dietary intake and disordered eating patterns have been linked to amenorrhea in a number of studies. A concept that has been developed supporting the link between dietary intake and amenorrhea is the so-called energy drain hypothesis. If caloric intake is too low, production of hormones such as estrogen and progesterone are moved lower on the list of priorities. These hormones may not be produced in amounts high enough to allow for menstruation to occur [14].

Endocrine and nutritional conditions can impair the delicate balance between bone formation and resorption, thus predisposing athletes to stress fractures. Oligomenorrheic or amenorrheic female athletes are at increased risk for developing stress, likely secondary to decreased estrogen levels and increased osteoclastic activity [15]. Stress fractures are also associated with lower fat intake, lower calorie intake, eating disorders, and body weight of <75 % ideal body weight. The female athlete triad (menstrual irregularity, inadequate caloric intake, and decreased bone mineral density) has been associated with increased susceptibility to stress fractures and may contribute to the increased stress fracture risk seen in female athletes and female military recruits compared with males performing the same activities [16]. High-intensity training may suppress menses, which may exacerbate these risk factors [17].

A recent pilot study indicated that female track and field/cross-country runners had an increased risk of developing stress fractures if body mass index (BMI) was less than 19. The authors of this case series found that female athletes with BMI of 19 or lower took significantly longer to return to unrestricted training and competition than those with a BMI above 19 [18].

### The male endurance athlete tetrad

Recent literature suggests that male runners may be predisposed to decreased bone mineral density. This has been shown to be the most notable in the lumbar spine and radius. The cause of this decreased density is most likely multifactorial. Inadequate caloric intake, decreased testosterone levels, and a genetic predilection are suspected of being the main culprits [19]. Decreased energy availability may be the key factor for low bone mineral density. Decreased testosterone levels have been shown to be present in males who participate in prolonged endurance events [19]. To prevent severe or irreversible effects of low BMD, it is necessary to assess the nutritional behaviors of male endurance athletes [20].

## High-risk stress fracture sites

Some stress fractures are affected by delayed or non-union because of insufficient blood supply to the region (Table 2). Proximal fifth metatarsal and tarsal navicular fractures are particularly difficult to heal because they occur within the vascular “watershed” region [21]. Other high-risk sites occur at locations of tensile stress on the cortical surface. Stress fractures at these sites have a predilection to progress to complete fracture, delayed union, non-union, and re-fracture, or have significant long-term consequences should they progress to a complete fracture [21, 22]. They typically carry a poorer prognosis if they have a delay in diagnosis. A delay in treatment may prolong the patient’s period of complete rest of the fracture site and potentially alter the treatment strategy to include surgical fixation with possible bone grafting [21, 22]. Due to their location on the tension side of the respective bones, these fractures possess common biomechanical properties regarding propagation of the fracture line. With delay in diagnosis or with less aggressive treatment, high-risk stress fractures tend to progress to complete fracture or non-union, require operative management, and recur in the same location [3, 21, 23].

**Table 2:** High-risk stress fracture sites [22]

Olecranon Scaphoid Tension side femoral neck Patella Anterior tibia Medial malleolus Talus Navicular 5th metatarsal proximal metaphysis Great toe sesamoids	

## Presentation and physical examination findings

**Fig. 1:**
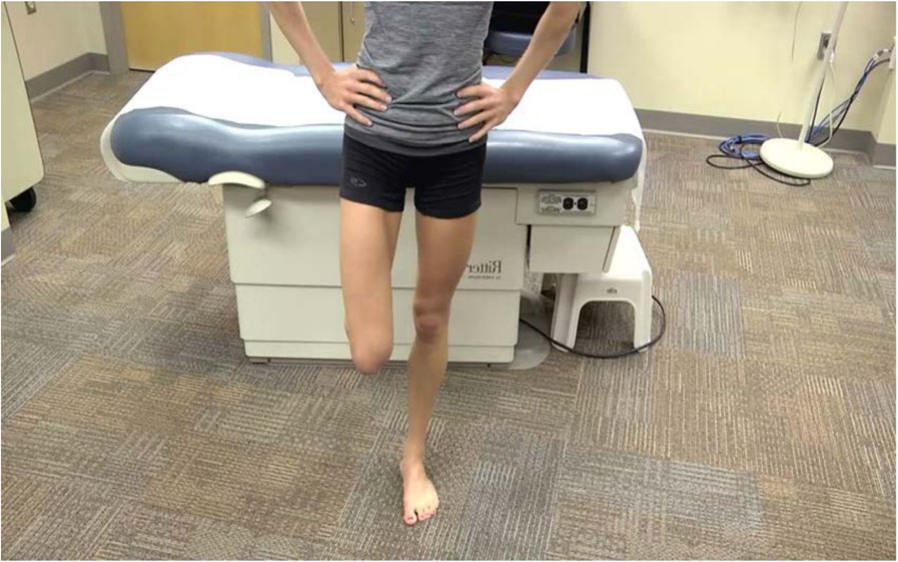
Athlete demonstrates a single-leg hop test. The athlete is asked to perform three hops in which the foot completely leaves the ground

Pain that is initially present only during activity is common in patients presenting with a stress fracture. Symptom onset is usually insidious, and typically, patients cannot recall a specific injury or trauma to the affected area. If activity level is not decreased or modified, symptoms persist or worsen [3, 17, 23]. Those who continue to train without modification of their activities may develop pain with normal daily activity and potentially sustain a complete fracture [24]. Physical examination reveals reproducible point tenderness with direct palpation of the affected bone site. There may or may not be swelling or a palpable soft tissue or bone reaction. Lower extremity stress fractures will commonly display reproduction of pain with single-leg hop testing (Fig. 1), log roll testing for injury of the femoral neck, fulcrum testing for the long bones, and tuning fork testing for occult fractures [4, 21, 24].

## Laboratory evaluation

Vitamin D deficiency has previously been discussed in this review. Other important laboratory values to obtain when treating male and female athletes with recurrent stress fractures include serum calcium and phosphate levels, parathyroid hormone (PTH), thyroid-stimulating hormone (TSH), alkaline phosphatase, albumin, and pre-albumin [4, 7, 23]. These tests are crucial for assessing nutritional status and healing potential. In female athletes, serum follicle-stimulating hormone (FSH), luteinizing hormone (LH), and estradiol levels are recommended to determine if an underlying endocrine condition or energy imbalance is contributing to decreased bone mineral density or recurrent injury [25].

## Imaging evaluation

### Radiography

**Fig. 2:**
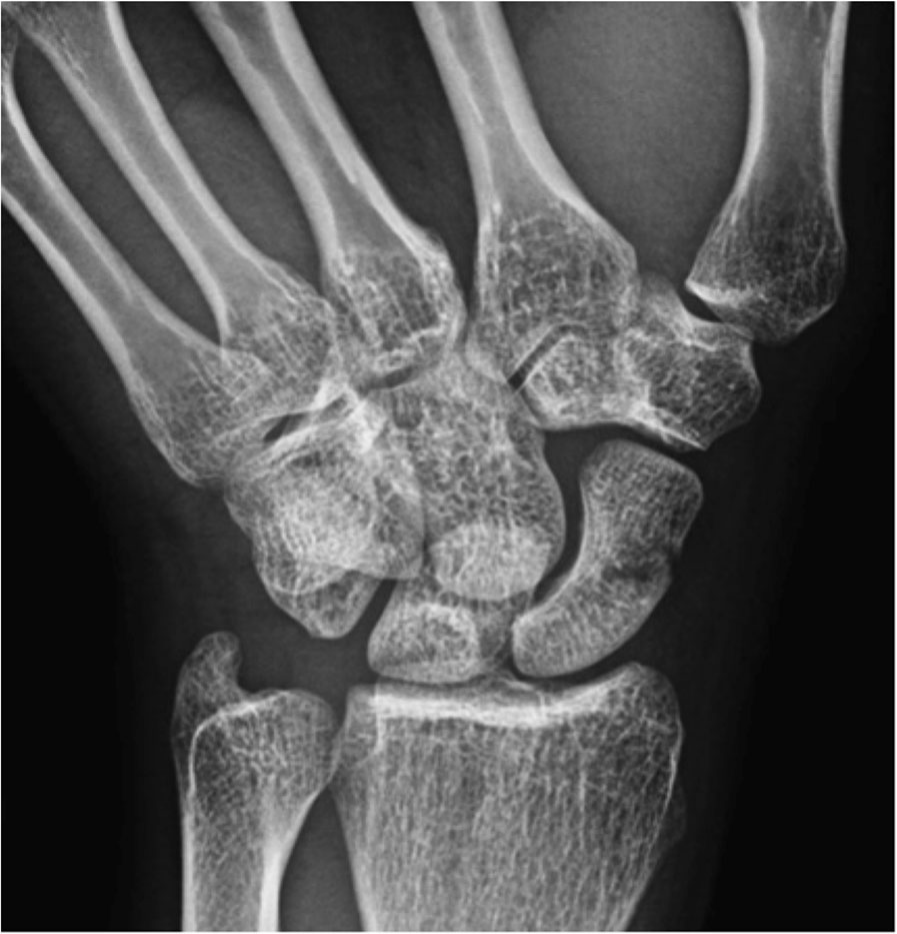
Scaphoid view radiograph of the left wrist in a gymnast with continued radial wrist pain demonstrating grade III scaphoid waist stress fracture

Two thirds of initial radiographs are normal early in the course of a stress fracture, but half ultimately prove positive once healing begins to occur making standard radiographs specific but not sensitive [26]. Even after healing has begun to occur, radiographic findings can be subtle and may be easily overlooked [26, 27]. Figure 2 demonstrates a radiograph of a subacute stress fracture of the scaphoid waist in a gymnast with chronic wrist pain.

### Bone scintigraphy

Bone scintigraphy had for many years been regarded as the gold standard for evaluating stress-induced injuries. Although recently supplanted by magnetic resonance imaging (MRI), it continues to be widely utilized in many situations [28]. Bone scintigraphy measures bone response to injury by depicting areas of increased osseous metabolism through the localization of radionuclide tracers, particularly Tc-99m-MDP [28]. The degree of uptake depends on the rate of bone turnover and local blood flow, and abnormal uptake may be seen within 6 to 72 h of injury [29]. Whole body bone scans can be performed with relatively low cost and have the advantage of being able to image the entire skeletal system at once. The sensitivity of bone scintigraphy is nearly 100 % [29]. The disadvantage of this technique is that the images may demonstrate for up to 2 years after the fracture site has become asymptomatic [28].

### CT

**Fig. 3:**
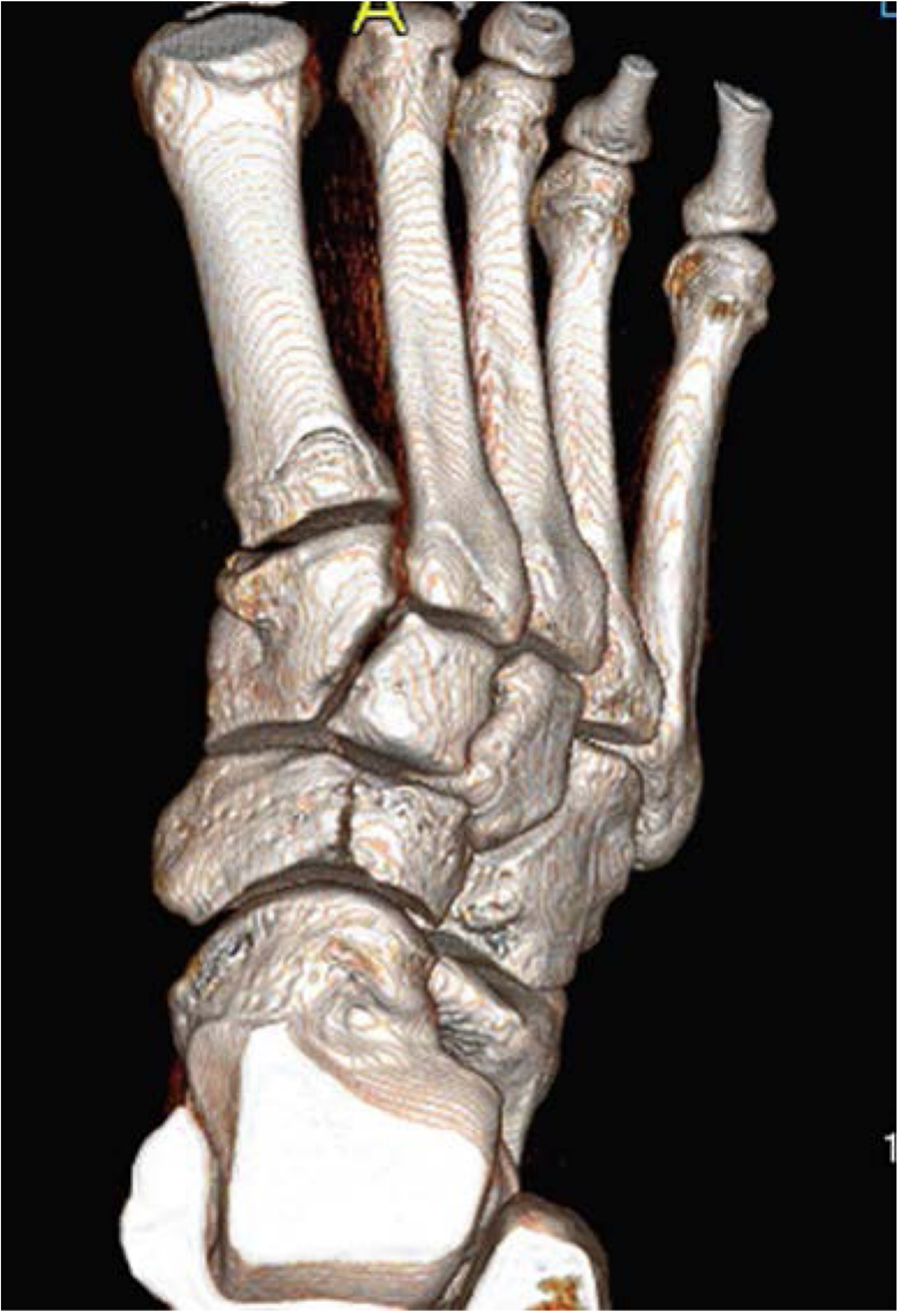
Three-dimensional CT scan of the right foot demonstrating grade III stress fracture of the central one third of the dorsal navicular

Computed tomography (CT) delineates the bone well and is useful when the diagnosis of a stress injury is difficult, particularly in the case of tarsal navicular stress fractures (Fig. 3) as well as linear stress fractures that may occur in the tibia [4, 27, 30, 31]. CT scanning is useful for demonstrating evidence of healing by clearly demonstrating the periosteal reaction and the absence of a discrete lucency or sclerotic fracture line [4, 27, 31]. It is also helpful in determining if the fracture is complete or incomplete.

### MRI

**Fig. 4:**
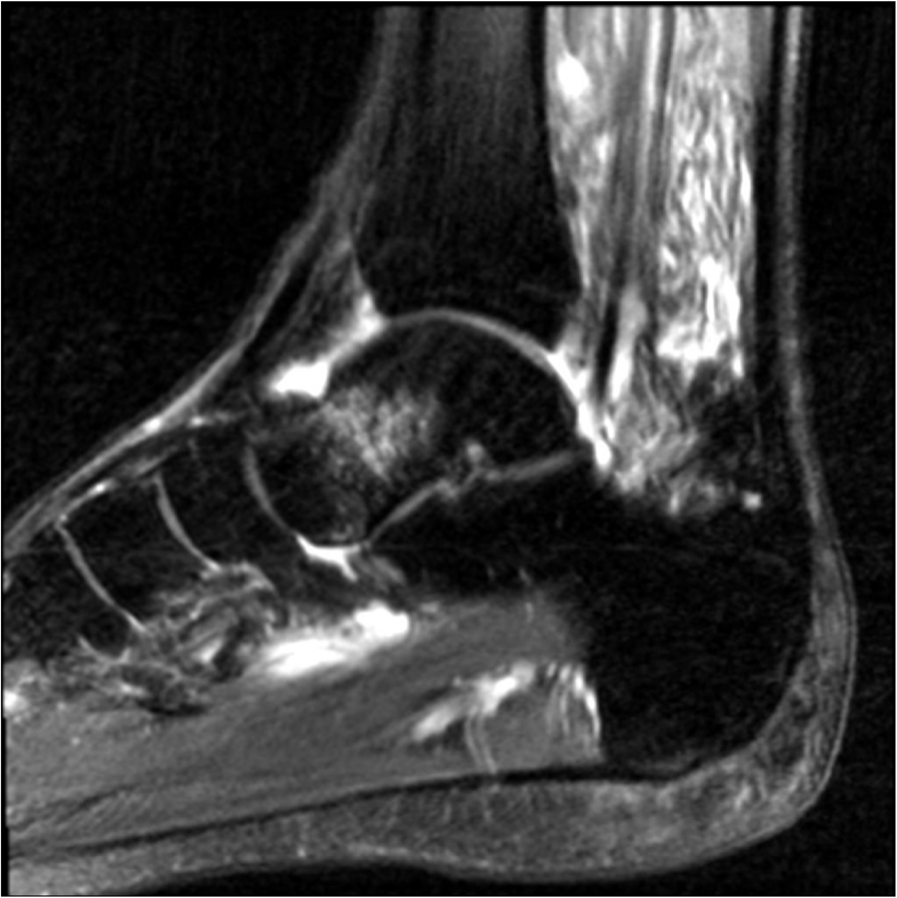
T2 sagittal MRI of the ankle demonstrating grade II stress fracture/stress reaction of the talar neck

MRI is an effective diagnostic technique in patients who show strong clinical manifestations of a stress fracture but have normal initial radiographs [32–34]. Like scintigraphy, MRI depicts changes in the bone and periosteum weeks before any radiographic abnormality develops [35]. The early stages of a stress fracture are characterized by focal hyperemia and bone marrow edema that correlates with the development of microfractures and osseous resorption (Fig. 4). Endosteal reactive changes, periostitis, and peri-osseous edema are important early observations on short-tau inversion recovery (STIR) or T2-weighted spin-echo images and are the characteristic of stress reactions [32, 35, 36]. The most common patterns of a fatigue stress fracture on MRI are a linear, uni-cortically based abnormality of low signal intensity surrounded by a larger, ill-defined region of marrow edema or a linear cortical abnormality with adjacent muscular or soft tissue edema. The presence of callus indicates a more chronic stress fracture.

MRI has comparable sensitivity to nuclear scintigraphy [37]. Specificity, accuracy, positive predictive value, and negative predictive value are all superior at 100, 90, 100, and 62 %, respectively [27, 32, 35]. Additionally, MRI has a distinct advantage by depicting the surrounding soft tissues, thus permitting concomitant evaluation of muscular, tendinous, or ligamentous structures [37, 38]. In the athletic population, injuries to any of these structures may mimic the symptoms of a stress fracture, which are sources that reduce the specificity of nuclear scintigraphic studies [39, 40].

### Classification/grading

In addition to knowing the classification of whether a stress fracture is high risk or low risk as determined by its anatomic site, the extent of the fatigue failure or “grade” of the stress fracture is preferred to completely describe the injury and make appropriate treatment plans [1, 41].

Recently, Kaeding and Miller have proposed a comprehensive descriptive system for stress fractures [1] (Table 3). This includes a grading scale for classifying the extent of structural failure from grade I to grade V. Grade I injuries are asymptomatic, usually incidental findings on imaging studies. Grade II injuries have imaging evidence of fatigue failure of bone, but no fracture line. Grade III injuries have a fracture line with no displacement, grade IV fractures are displaced, and grade V stress fractures are chronic having gone onto non-union. The system has demonstrated high levels of inter- and intra-observer reliability among Sports Medicine care providers and has been shown to be predictive of time to return to sports [1, 18].

**Table 3:** Kaeding-Miller stress fracture classification system [1]

Grade	Pain	Radiographic findings (CT, MRI, bone scan, or X-ray)
I	−	Imaging evidence of stress FX No fracture line
II	+	Imaging evidence of stress FX No fracture line
III	+	Non-displaced fracture line
IV	+	Displaced fracture (>2 mm)
V	+	Non-union

## Optimizing the biologic, biomechanical, and psychological environment

The immediate goal of treatment of a high-risk stress fracture is to avoid progression and achieve complete healing [19]. Ideally, as the fracture is healing, the athlete may work to avoid deconditioning while minimizing the risk of a significant complication of fracture healing [4, 7, 17, 23]. While over-treatment of a low-risk stress fracture may result in unnecessary deconditioning and loss of playing time, under-treatment of a high-risk injury puts the athlete at risk of significant complications such as delayed healing, incomplete healing, and refracture [21, 22]. In this case, relative rest may be achieved with alternative training options such as aquatic training which may include an aquatic treadmill or suspended treadmill training.

The presence of a visible fracture line on a plain radiograph in a high-risk stress fracture should prompt serious consideration of operative management. If an incomplete fracture is present on plain films with evidence of fracture on MRI or CT in a high-risk location, immobilization and strict non-weight bearing is indicated [21]. Worsening symptoms or radiographic evidence of fracture progression despite non-operative treatment is an indication for surgical fixation [3, 4].

**Fig. 5:**
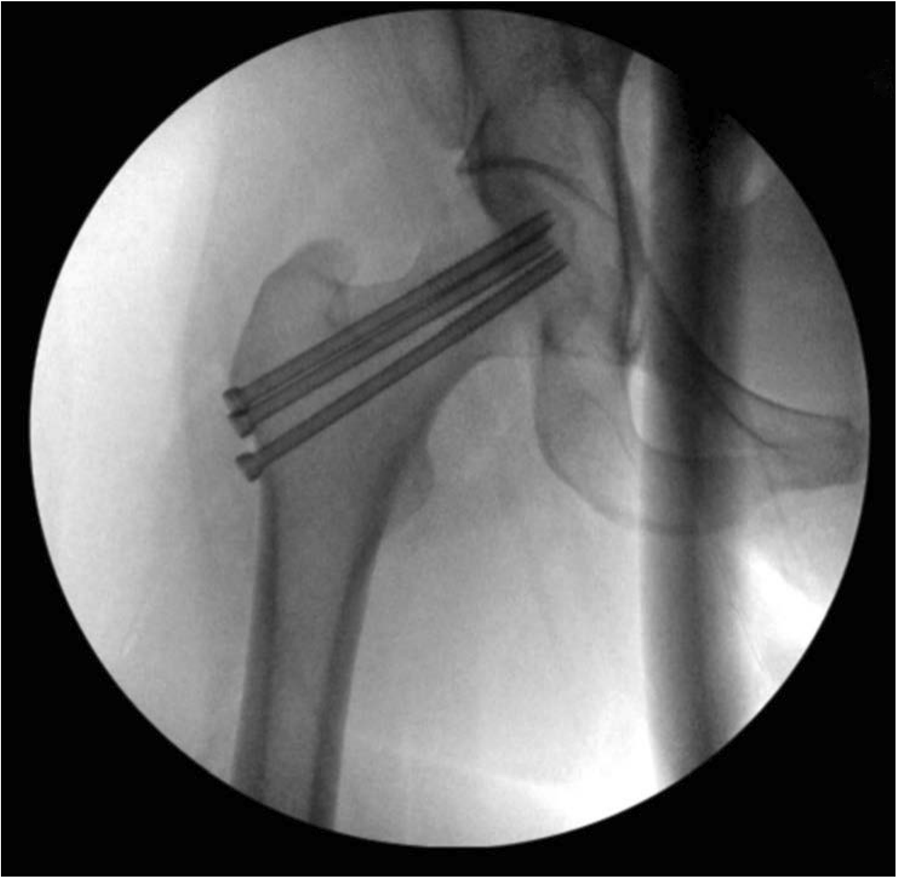
Intraoperative fluoroscopic radiograph of the right hip demonstrating screw fixation of a femoral neck stress fracture

**Fig. 6:**
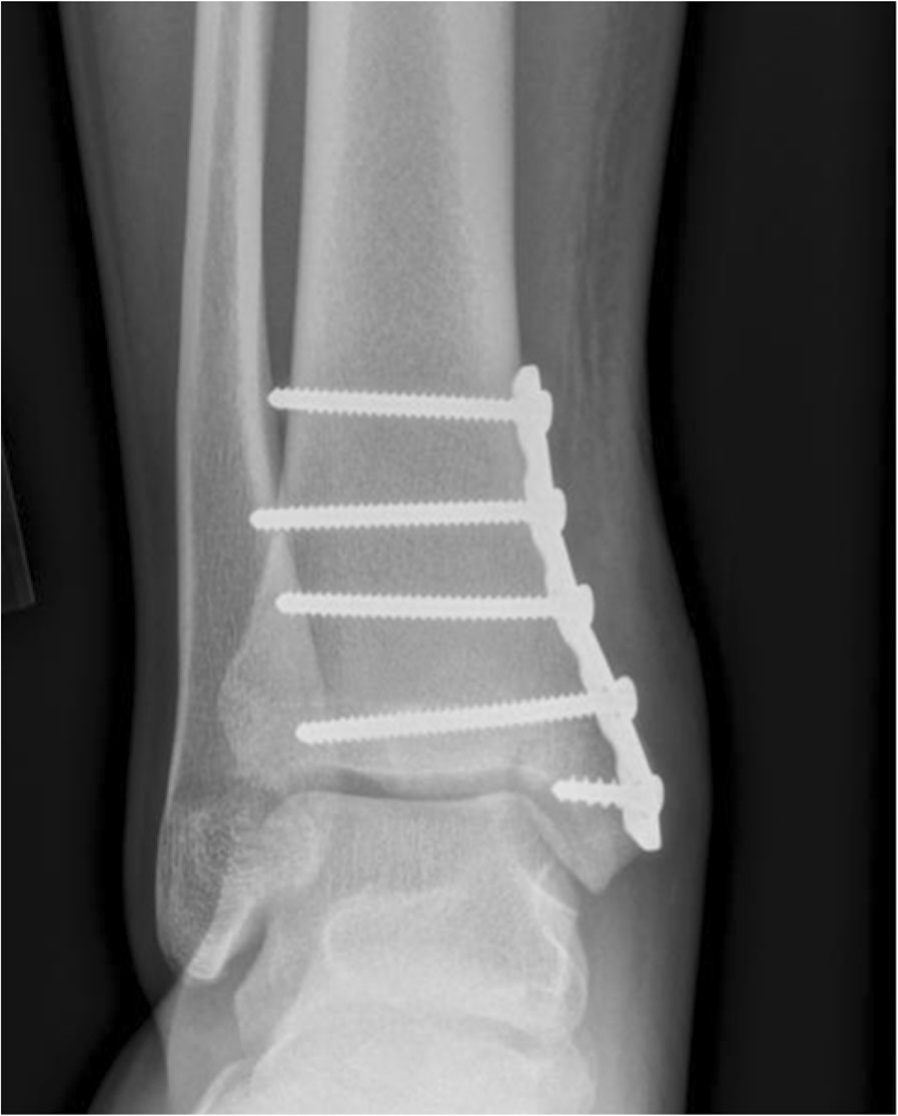
Post-fixation radiograph of a collegiate soccer player with medial malleolar stress fracture

All complete fractures at high-risk sites should receive strong consideration for surgical treatment. Surgical fixation should be considered for high-risk stress fractures for several reasons. These include expediting healing of the fracture to allow earlier return to full activity as well as to minimize the risk of non-union, delayed union, and re-fracture [4, 7, 21, 22]. Finally, surgical intervention may be necessary to prevent catastrophic fracture progression such as in the case of the tension-side of the femoral neck (Fig. 5) or medial malleolar stress fracture (Fig. 6).

## Return to sports decision-making

Generally in athletes, return to play should only be recommended after proper treatment and complete healing of the injury. Shared decision-making between the physician, athletic trainer, coach, and athlete is recommended. Because of the significant complications associated with progression to complete fracture, it is not recommended that an individual be allowed to continue to participate in their activity with evidence of a high-risk stress fracture [7, 21, 23]. Return to play decision-making for a low-grade injury at a high-risk location should be predicated on the patient’s compliance level, healing potential, and risk of fracture propagation. A key difference between a low-grade stress fracture at a high-risk location versus a low-risk location is that with the low-risk site, the athlete or patient can be allowed to continue to train, whereas the high-risk site needs to heal prior to full return to activity [3, 4, 17].

**Fig. 7:**
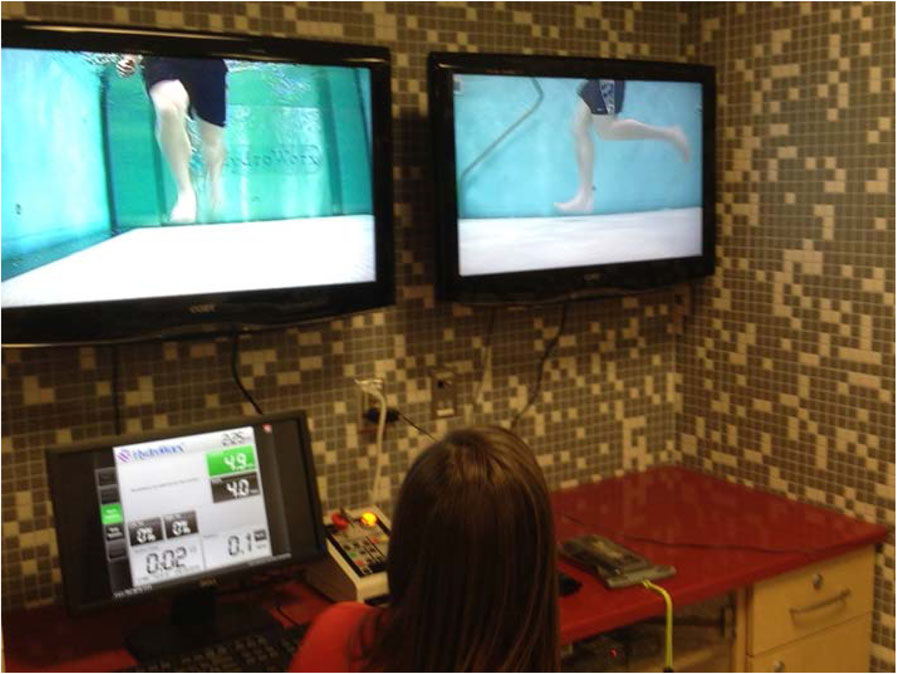
Running gait evaluation performed via aquatic treadmill in a male distance runner

A recent study of Division I collegiate track and field athletes indicated that expected to return to unrestricted training and competition ranged from 11 to 17 weeks [18]. Time to return varied linearly dependent on severity grade based on the Kaeding-Miller classification system. Criteria for allowing an athlete to return should include complete resolution of symptoms with activities of daily living, radiographic evidence of healing, no tenderness to palpation at the injury site, and optimization of the athlete’s nutritional, biomechanical, hormonal, and psychological status [4]. Recently, dual-energy X-ray absorptiometry (iDEXA) has been suggested to assure optimal lean to non-lean mass has been established and is currently under investigation to determine its ability to decrease future stress fracture risk. Training progression includes resistance training to optimize muscle mass along with the use of low-impact training options. Stationary biking, elliptical trainer, aquatic treadmill (Fig. 7), and suspended treadmill (Alter G) are utilized to maintain fitness as land running and participation in the causative activity are gradually increased.

## Preventing recurrence

Prevention is the ideal treatment of bone stress injuries. An assessment of the athlete’s risks should be made at pre-participation evaluations, especially in those with a history of previous stress fractures. Correction of amenorrhea in females and calcium and Vitamin D supplementation is recommended in addition to general nutritional optimization. If biomechanical abnormalities are encountered, the use of appropriately designed orthotic devices should be considered as an initial corrective measure. However, a running gait analysis to correct running form and biomechanics may be necessary to prevent future injuries. Additionally, bone density with body composition evaluation (iDEXA) may be helpful in individuals with recurrent bony stress injuries.

Keys to preventing stress fractures include appropriate equipment, technique, and coaching, optimization of nutrition and hormonal status, and optimization of body composition with a balanced lean mass to non-lean mass ratio. Cross-training and alternative training using devices such as an aquatic treadmill or anti-gravity treadmill allows running athletes to maintain cardiovascular fitness and running form while minimizing ground reaction forces to the lower extremity. The importance of adequate rest and recovery from training and competition to allow for healing of microtrauma to bones cannot be understated. In an era of continued single-sports specialization, off seasons and varying the training regimen and training environment are paramount for preventing stress injuries and other overuse conditions in endurance athletes.

## Conclusions

High-risk stress fractures are common injuries particularly in endurance athletes and military recruits. Effective management of these injuries should employ a holistic approach and be individualized to the patient or athlete. It is necessary to take into account the injury site (low vs. high risk), the fracture grade (extent of microdamage accumulation), the individual’s competition level, and their risk profile. Healing and prevention requires optimization of the healing environment including the athlete’s nutritional, hormonal, biomechanical, and psychological status. Aggressive treatment is required for stress fractures at high-risk sites. This often employs complete rest, immobilization, and surgical stabilization to prevent fracture progression, displacement, or non-union.

## Abbreviations

BMI: Body mass index
CT: Computed tomography
iDEXA: Dual-energy X-ray absorptiometry
FSH: Follicle-stimulating hormone
LH: Luteinizing hormone
MRI: Magnetic resonance imaging
PTH: Parathyroid hormone
STIR: Short-tau inversion recovery
TSH: Thyroid-stimulating hormone
